# Airglow-imager based observation of possible influences of subtropical mesospheric gravity waves on F-region ionosphere over Jammu & Kashmir, India

**DOI:** 10.1038/s41598-021-89694-3

**Published:** 2021-05-13

**Authors:** T. K. Ramkumar, Manzoor Ahmad Malik, Bilal Ahmad Ganaie, Aashiq Hussain Bhat

**Affiliations:** 1 National Atmospheric Research Laboratory, DOS, Govt. of India, Gadanki, Andhra Pradesh 517112 India; 2 Department of Physics, University of Kashmir, Jammu and Kashmir, Srinagar, 190006 India

**Keywords:** Environmental sciences, Space physics

## Abstract

As a joint research collaboration between the National Atmospheric Research Laboratory (NARL), and the University of Kashmir (KU), NARL installed an all-sky airglow CCD imager (with centre wavelengths of 630 nm, 557.7 nm [2 nm band widths] and 840 nm [150 nm wide band with blocking notch at 866 nm to avoid the contamination of molecular oxygen emissions]) in the University campus in Srinagar (75°E, 34°N, geographic), Jammu and Kashmir, India (western Himalayan region). To understand the upper atmospheric dynamics and ionospheric electrodynamics and their associated physical coupling mechanisms, the imager observes airglow emissions of OH molecules (~ 85 km height; 840 nm) and atomic oxygen occurring at the heights of ~ 97 km (557.7 nm) and ~ 250 km (630 nm). Airglow observations in Kashmir commenced in the night of August 11, 2017 and the present work reports on the characteristics of first-time observation of Medium Scale Travelling Ionospheric Disturbances (MSTIDs with horizontal wavelengths of ~ 100–300 km) over Kashmir region during 20:30—22:30 IST (Indian standard time) on August 15, 2017 (India independence day). Initially, the phase front of MSTIDs was aligned along the north-west and south-east direction and moved at ~ 57 m/s towards the south-west direction and finally the westward direction by aligning along the meridian before they disappeared. Along with SAMI-3 ionospheric model simulations, simultaneous multiwavelength airglow observations indicate that secondary gravity waves generated due to dissipation of upward propagating mesospheric gravity waves in the heights of ~ 85–95 km would have contributed to the generation of MSTIDs in the F region ionospheric plasma through electrodynamical coupling between the E and F region (Perkins instability) ionosphere.

## Introduction

Medium Scale Travelling Ionospheric Disturbances (MSTIDs) can be induced by atmospheric gravity waves generated by wind flow over mountains, wind shears and jet streams, auroral Joule heating effects, lower atmospheric convection and many other sources^1–8^. MSTIDs can be identified as mesoscale wave-like perturbation of the ionospheric plasma with horizontal wavelengths of several hundred kilometers, period of 15–60 min, and horizontal velocity of 100–250 m/s often observed in the mid latitude F-region ionosphere^9,10^. Using the data of 350 Global Positioning System (GPS) receivers of ionospheric total electron content obtained over Southern California, Kotate et al.^5^ studied in detail the statistical characteristics of MSTIDs. They categorized MSTIDs into (1) daytime MSTIDs which can be generated due to atmospheric gravity waves in the thermosphere mainly in winter and equinoxes and propagate mostly south eastward, (2) night time MSTIDs occurring frequently in summer and propagating south westward, which is consistent with the idea of polarization electric fields playing an important role in generating night time MSTIDs, (3) dusk time MSTIDs occurring frequently in summer and propagating north westward. Dusk time MSTIDs are thought to be caused by atmospheric gravity waves originating from the sunset terminator as the wave fronts are almost parallel to the sunset terminator. Airglow images obtained using high-sensitive CCD cameras have revealed that night time MSTIDs normally propagate southward^11–14^, which cannot be explained by the classical theory of gravity waves^15,16^. Normally, day time/night time MSTIDs occur frequently in winter/June solstice in the Japanese and Australian longitudinal sector and near December solstice in the European longitudinal sector^5,14,17,18^. Earlier studies over Japan report that there is a good match between horizontal two-dimensional maps of total electron content perturbations (TECp) as determined by GPS receivers and F region ionospheric 630 nm emissions^19,20^. Similar observations of both day and night time MSTIDs are also reported for the North American region^5,7^. In addition, night time F region field-aligned irregularities (FAIs) as observed by the middle and upper atmosphere (MU) radar in Japan also show high correlation with MSTIDs activity, both propagated south-westward with same velocities^20^. Night time MSTIDs occur mainly in the bottom side of the F region where the peak emission rate of 630-nm airglow occurs ^13,19^. Apart from observations of travelling ionospheric disturbances (TIDs) using airglow cameras and dual-frequency GPS receivers, studies on TIDs are also reported using high frequency (HF) radar observations^21–23^. From the analyses of HF radar observations at three different heights in the F region ionosphere, Chum and Podolska^21^ experimentally analysed propagation of GWs in three dimensions (3D).

Some TIDs are identified as Travelling Wave Packet TIDs (TWPTID) which is associated with atmospheric acoustic-gravity waves. GPS based study of TWPTIDs indicates that they appear as quasi periodic wave packets with Gaussian envelopes of width ∼1 h modulated with periods of few tens of minutes^1^. Based on space based (COSMIC also known as FORMOSAT‐3) observation of ultra violet emissions from the ionosphere in conjunction with the Very Large Array (VLA) radio telescope, located near Socorro, NM, Dymond et al.^24^ reported the observation of MSTIDs. They observed the MSTIDs as waves with 23.8, 11.9, and 10.6 min periods having speeds > 200 m/s and traveling faster than typical night time MSTIDs speeds of ∼100–150 m/s which are slower than the speed of sound in the thermosphere (∼ 400–800 m/s) and are within the speed range associated with MSTIDs^25^. Further, their VLA observations indicate the presence of MSTIDs with amplitudes of 0.14, 0.05, and 0.07 TECU (total electron content unit = 10^16^ electrons/m^2^) and with the corresponding horizontal wavelengths of 239, 188, and 162 km. Using ground-based GPS receivers in the low latitude region of Taiwan, earlier Lee et al.^26^ detected night time MSTIDs, with wave fronts aligned along the northwest-southeast direction and with wavelength of ~ 500 km, moving south westward to latitude of 20.5°N at the horizontal velocities between 100 and 160 m/s. Further, their observations indicated that MSTIDs play important role in the generation of low-latitude F-region ionospheric plasma irregularities.

Strong polarization electric fields found inside the structure of night time MSTIDs indicate that Perkins instability could play a major role in the generation of night time MSTIDs^11,27–29^. The Arecibo incoherent scatter radar also detected intense polarization electric field associated with night-time mid-latitude F-region ionospheric MSTIDs^16^. The earlier classical theory of influences of atmospheric gravity waves on the generation of TIDs did not incorporate the effect of disturbed polarization electric field generated in the F region ionosphere^30^. Perkins instability could successfully explain the normal observation of northwest–southeast alignment of density perturbations associated with night time MSTIDs^31^. However, it predicts the propagation direction of MSTIDs in the north-eastward rather than the south-westward direction and the generated perturbation electric fields aligned along the MSTID wave fronts could cause them to move south westward^5^. Various atmospheric and ionospheric phenomena like atmospheric gravity waves^10^, electrodynamic coupling between F- and E regions^32^ and between two hemispheres^14,33^ is considered as an important seeding mechanism of the Perkins instability. The restriction in the southward propagation of MSTIDs beyond ~ 20°N is due to higher electron densities in the low latitude regions as they can impede the southward advancement of atmospheric gravity waves (seed of the wave-like structure of MSTIDs) through the ion-drag effect^14,30,34,35^. The complex meso-scale structures of MSTIDs indicate the existence of an unknown interaction between the neutral atmosphere and the ionized plasma in the mid latitude ionosphere.

Given the complex scenario of MSTIDs, as a collaborative research effort between NARL and the Dept. of Physics of University of Kashmir, Srinagar, Jammu and Kashmir, India, NARL has installed one all-sky airglow imager in the University campus to study the characteristics of mesospheric wave dynamics and the ionospheric electrodynamics in the Indian subtropical region. Since the Kashmir region is located in the western Himalayan valley region, it is expected that the atmospheric winds associated with subtropical jets can induce different kinds of atmospheric gravity waves upon hitting the western Himalayan hills from the west. The imager is operating at the centre wavelengths of (1) 630 nm [atomic oxygen emission with peak at ~ 250 km], (2) 557.7 nm [atomic oxygen emission with peak at ~ 97 km; 2 nm band widths] and (3) 840 nm [OH emission (peak at ~ 86 km) with wide 200 nm band width and notch filter at 868 nm to get rid of contaminating molecular oxygen emissions from the height of about 95 km). This paper delves on the linkage between the atmospheric gravity waves observed at the height of ~ 86 km and the travelling ionospheric disturbances namely MSTIDs detected at the height of ~ 250 km.

## Airglow CCD imager data processing and SAMI3 ionosphere model

Using a circular medium format F/4 Mamiya fish-eye-lens (24 mm focal length), the airglow CCD imager consists of a set of plano-convex lens in the front optics to make parallel the rays of the collected light from space. These parallel rays are allowed to pass through temperature controlled Fabry–Perot etalon (interference filters) which is normally maintained at 25 °C. Filters are located inside a filter wheel that can accommodate up to six 3-inch diameter filters each with thickness of a quarter of an inch and they have 50–90% transmission coefficients. Filters corresponding to the centre wavelengths of 630 nm (O[^1^D]) and 557.7 nm (O^1^S) have a narrow band width of about 2 nm and the OH 840 nm filter is a 720–920 nm wide band filter with blocking notch at ~ 868 nm to get rid of the contaminating molecular oxygen emissions. Separate narrow band filters with centre wavelengths of 866 and 868 nm are also fitted in the filter wheel. At near infrared (NIR) wavelengths, etaloning effects in the CCD camera are prominent and the data of which are not presented here as it requires further treatment of received emission signals. The optical rays after passing through the filters are allowed to pass through a relay optics system and finally they are collected on to a CCD camera (PIXIS, Princeton Instruments) which contains a back illuminated e2vCCD47-10 chip (ActonPixis1024B) with 1024 × 1024 square pixels each of which is having a size of 13.3 µm,100% fill factor and 16-bit depth. The camera is cooled to − 70 °C to minimise dark noise. In the present work, pixels are 2 × 2 bin averaged and hence finally the png format images generated will have 512 × 512 array of 16-bit digitized data values. The experiments were conducted by setting the scanning time of 100 s for the 630 nm and 557.76 nm filters and 10 s for the 840 nm filter as it is a wide band filter through which more intense signals will propagate. The generated data images are then utilized for further analyses and the results are presented. Further details of the instrument and data, methodology of extraction of geographical coordinate information of various airglow emissions occurring at different heights can be found elsewhere^36–38^. The present work adopted the image processing algorithms as mentioned in Alok et al.^36^.

### Imager data processing

Using the relationship between the coordinates of the fish-eye-lens of the front optical system of the imager (zenith angle θ and azimuthal angle φ) and the geography of the imager site for a particular height in the atmosphere (horizontal distance r from the centre of the site and the azimuthal angle), the obtained raw images are un-warped to determine the geographical coordinates of particular interests of atmospheric and ionospheric information^38^.

$$ {\text{r}} = {\text{R}}_{{\text{E}}} \times \alpha $$

$$\alpha = \theta - {\text{sin}}^{ - 1} \left( {{\text{R}}_{{\text{E}}} \times {\text{sin}}\,{\theta}/\left( {{\text{R}}_{{\text{E}}} + {\text{h}}_{{{\text{ag}}}} } \right)} \right)$$

where θ is the zenith angle, R_E_ is the radius of the Earth and h_ag_ is the altitude and r is the horizontal radial distance from the centre of the image (site location) to the point of interest corresponding to the zenith angle (geographical spatial information) of the airglow emission layer.

With this information of geographical coordinates of all the pixels of the observed image data and time sequenced images, the horizontal wavelength, period and phase speed and direction of moving phase fronts of atmospheric gravity waves or ionospheric plasma irregularity waves manifested in intensities of airglow emissions can be determined. Further, the phase speed of waves can be obtained from keograms by choosing a line along a particular phase front and calculating its slope from both the zonal and meridional keograms. This gives us the two components of phase velocities. e.g. for a SW moving wave we get southward component from meridional keogram and westward component from the zonal keogram. Then the resultant phase velocity of the wave event is given by

$${\text{v}}_{{\text{p}}} = \surd ({\text{v}}_{{\text{m}}}^{2} + {\text{v}}_{{\text{z}}}^{2}) ,$$

where v_m_ = meridional phase velocity component and v_z_ = zonal phase velocity component.

It is here to be noted that in the case of two-dimensional (2D) analysis of CCD imager observation of wave propagation in horizontal plane, the determined phase velocities are only apparent velocities as they are determined in the Earth reference frame and they depend on the elevation angles of wave vectors in 3D. The real velocities (corrected for curvature of space at a particular height of observation) can only be determined from the 3D analysis of wave propagation.

By knowing the period from time series of images, we can easily determine the horizontal wavelength from the knowledge of horizontal phase velocity. Horizontal wavelengths can also be determined directly from the images by noting the geographical information of successive dark or white bands in the zonal and meridional directions.

Since the optical system can introduce nonuniformities in the distribution of recorded airglow emission intensity across all the pixels, firstly it needs to be corrected (flat field correction) before proceeding for further analyses. Using imager calibration data for each of the wavelengths of optical filters supplied by Keo-Scientific (manufacturer of the imager), enough care has been taken to remove the non-uniformities in the present work. As a result, the intensity values are given in SI units as Watt sr^−1^ m^-2^ which can be converted in to rayleigh units through the Eq. ^39^.

For example,

$$ {\text{wavelength}},\,\lambda = \,630\,{\text{nm}} = 0.63\,\mu {\text{m}} $$

$$ 1\,{\text{ rayleigh}} = 10^{ - 9} /2\pi \lambda \,{\text{W}}\,{\text{sr}}^{ - 1} \,{\text{m}}^{ - 2} = 10^{ - 3} /2\pi 0.63\,{\text{W}}\,{\text{sr}}^{ - 1} \,{\text{m}}^{ - 2} = 0.25 \times 10^{ - 3} \,{\text{W}}\,{\text{sr}}^{ - 1} \,{\text{m}}^{ - 2} $$

$$ {\text{radiance}},\,L\, = \,0.01\,{\text{W}}\,{\text{sr}}^{ - 1} \,{\text{m}}^{ - 2} \,\left( {{\text{typical}}\,{\text{ from}}\,{\text{ Fig}}. \, \,2 \, \,{\text{of}}\,{\text{ the}}\,{\text{ present}}\,{\text{ work}}} \right) $$

$$ {\text{L}} = 0.01\,{\text{W}}\,{\text{sr}}^{ - 1} \,{\text{m}}^{ - 2} = 10 \times 10^{ - 3} \,{\text{W}}\,{\text{sr}}^{ - 1} \,{\text{m}}^{ - 2} = 40\,{\text{ rayleigh}} $$

Apart from this non-uniformity, for a particular wavelength, airglow intensity decreases for the off-zenith rays approaching the front optics from the side of the lens compared to the zenith rays. However, at low elevation angles in the sides of fish eye lens, thicker airglow emission layer leads to increase in intensity (Van Rhijn effect) and thus partially compensates for the loss due to oblique incidence of the rays. If we remove (say few hours of observation on a particular day) all pixels-averaged signal from the original raw signal of each and every pixel in a particular frame, the remaining other nonuniformities also will be significantly removed. Similar methodology is adopted in the present work and nonuniformities are largely removed except for strong signals associated with stars and galaxies, which can be easily distinguished from the atmospheric features.

Atmospheric wave motions and their essential wave parameters like horizontal wavelength, phase speed and direction are determined by identifying their bright bands (intense airglow emissions associated with crest of wave motions) and their spatial separations as well as their time evolutions in time series of images. Wave front moving direction can easily be identified in the un-warped images which contain geographical coordinate information at a particular height corresponding to particular wavelength of airglow emission associated with that height region. Since the gravity wave phase speed is determined by noticing the time movement of high intense bands associated with crest of gravity waves in space (geographical coordinate mapping in images), errors in the calculation of wave speed arises due to the time evolving diffusion of these intense bands that have sizeable widths in space. Taking this into account the roughly estimated errors in wave speed and wavelength are provided in closed brackets along with actual speed values wherever mentioned in the remaining text.

### SAMI3 ionosphere model description

In a unique, non-uniform, non-orthogonal and fixed grid, the Naval Research Laboratory (NRL) ionospheric plasma model SAMI3^40^ is a three-dimensional global model based on its earlier two-dimensional model SAMI^41,42^. With lower boundary at 85 km height, the SAMI3 calculates these ions densities: H^+^, He^+^, N^+^, O^+^, NO^+^, N_2_^+^ and O_2_^+^ ions in the heights of 85–20,000 km and it is well suited for the studies of MSTIDs also as its updated version includes the information of mesospheric gravity waves that lead to the generation of MSTIDs^40^. Further SAMI3 solves electron and ion temperature equations for H^+^, He^+^, and O^+^ ions by including ion inertia in the ion momentum equation along the geomagnetic (offset and tilted dipole) field lines. It offers modelling of the ionospheric plasma from hemisphere to hemisphere by employing ionospheric flux tube model. The information of vertical and zonal drifts of the ionospheric plasma due to **E x B** drifts associated with zonal (vertical drifts) and meridional (zonal drifts) electric fields is also included. While NRLMSISE00 model provides information of neutral composition and temperature, HWM model provides the neutral wind information as background input neutral atmospheric parameters for running the SAMI3 full physics model. More detailed information of the SAMI3 model data can be obtained from the website https://ccmc.gsfc.nasa.gov/models/modelinfo.php?model=SAMI3. For the present work, we used SAMI3 1.00.1version with 00 UT of 15 August 2017 as the initial condition. As the initial condition is more than 13 h before the onset of MSTIDs in the ionosphere, there is no question of doubting the relevance of the results of the SAMI 3 model while comparing them with CCD imager observations. It is 0.15° resolution in the latitudes and longitudes and it is 1.3 and 8.3 km height resolutions for the Figs. 11 and 10 respectively.

## Observations and results

**Figure 1:**
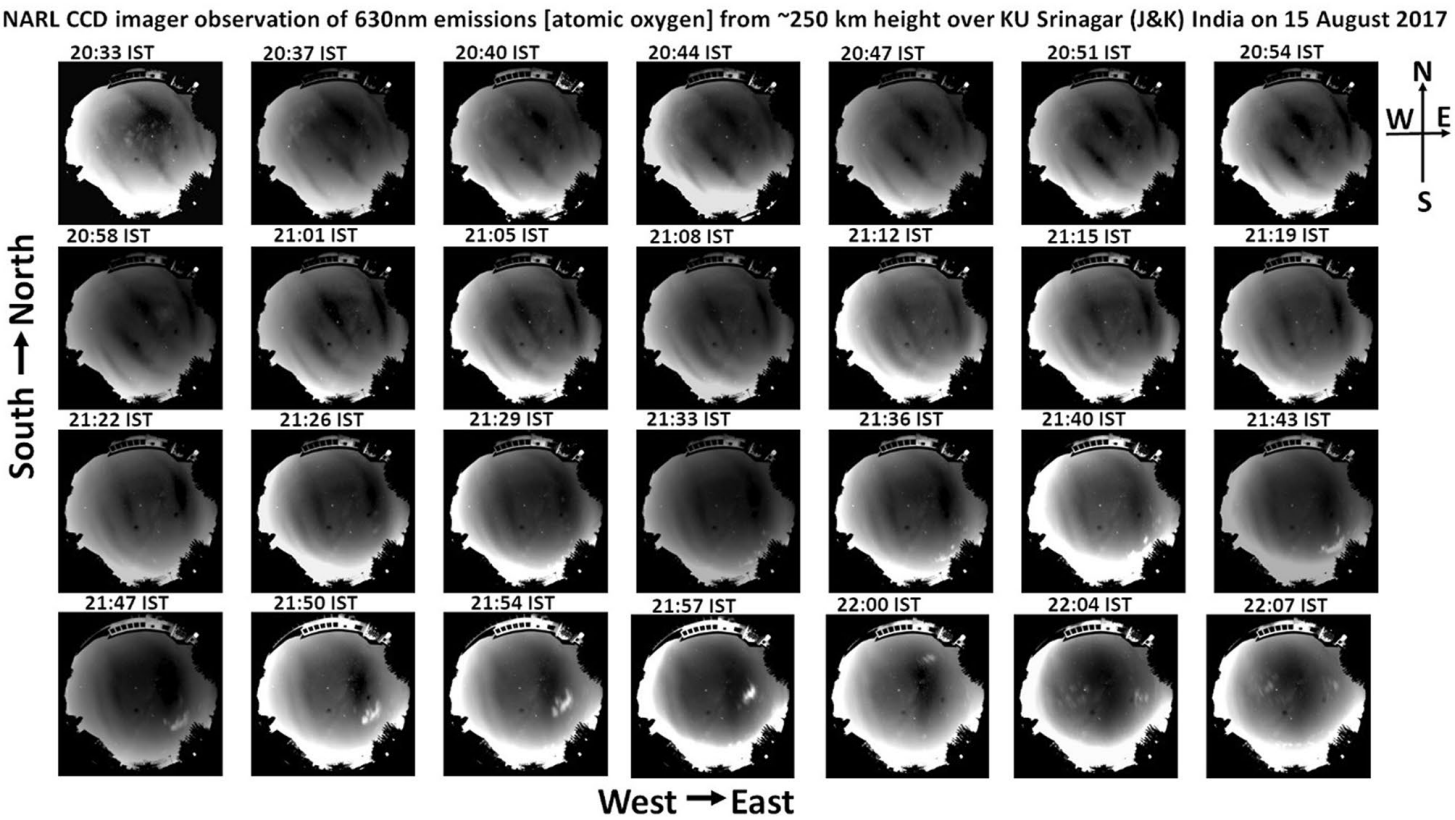
Shows 28 panels (scans) of all-sky airglow images taken with NARL CCD imager at 630 nm (2 nm band width) for atomic oxygen emissions at ~ 250 km height in the campus of University of Kashmir (KU), Srinagar, Jammu and Kashmir, India on 15 August 2017. The scan time is 100 s and the time interval of the scans is about 4 min.

Figure 1 shows the NARL all-sky airglow imager observations of 630 nm emission, by atomic oxygen at the height of about 250 km in the F region ionosphere over the Indian subtropical station of University of Kashmir (75°E, 34°N, geographic; magnetic latitude ~ 26 N; ~ 4000 m above mean sea level), Srinagar, Jammu and Kashmir, on 15 August 2017. In total, there are 28 panels of figures, each of which is obtained for an individual scan period of 100 s starting from 20:33 IST (Indian Standard Time = UT + 5:30 h) onwards and the time interval between two panel is about 4 min. It can be noticed that the panel at 20:33 IST shows diffused dark bands and in the subsequent panels the bands appear moving towards the south-west direction. The bands (phase fronts) are aligned along the north-west and south-east directions and the normal to these bands move towards the south-west direction. With time, multiple bands arrive in the field of view of the imager and move towards the south-west direction. There are three dark bands easily visible until 21:57 IST and afterwards only two bands are visible with the last band appearing at further distance from its nearby band compared to other inter-band distances. It can also be noted that the bands start to align along the north–south direction (meridian) as time progresses. At 21:57 IST, the bands are aligned almost parallel to the North–South direction. The last wider dark band, aligned along the meridian, continues to move towards the west at 22:00 IST. After reaching the centre of the image (over head of Srinagar), the dark band widens with time and at 22:07 IST, the dark band expanded in such a way that the whole image appears dark with no signatures of movement.

**Figure 2:**
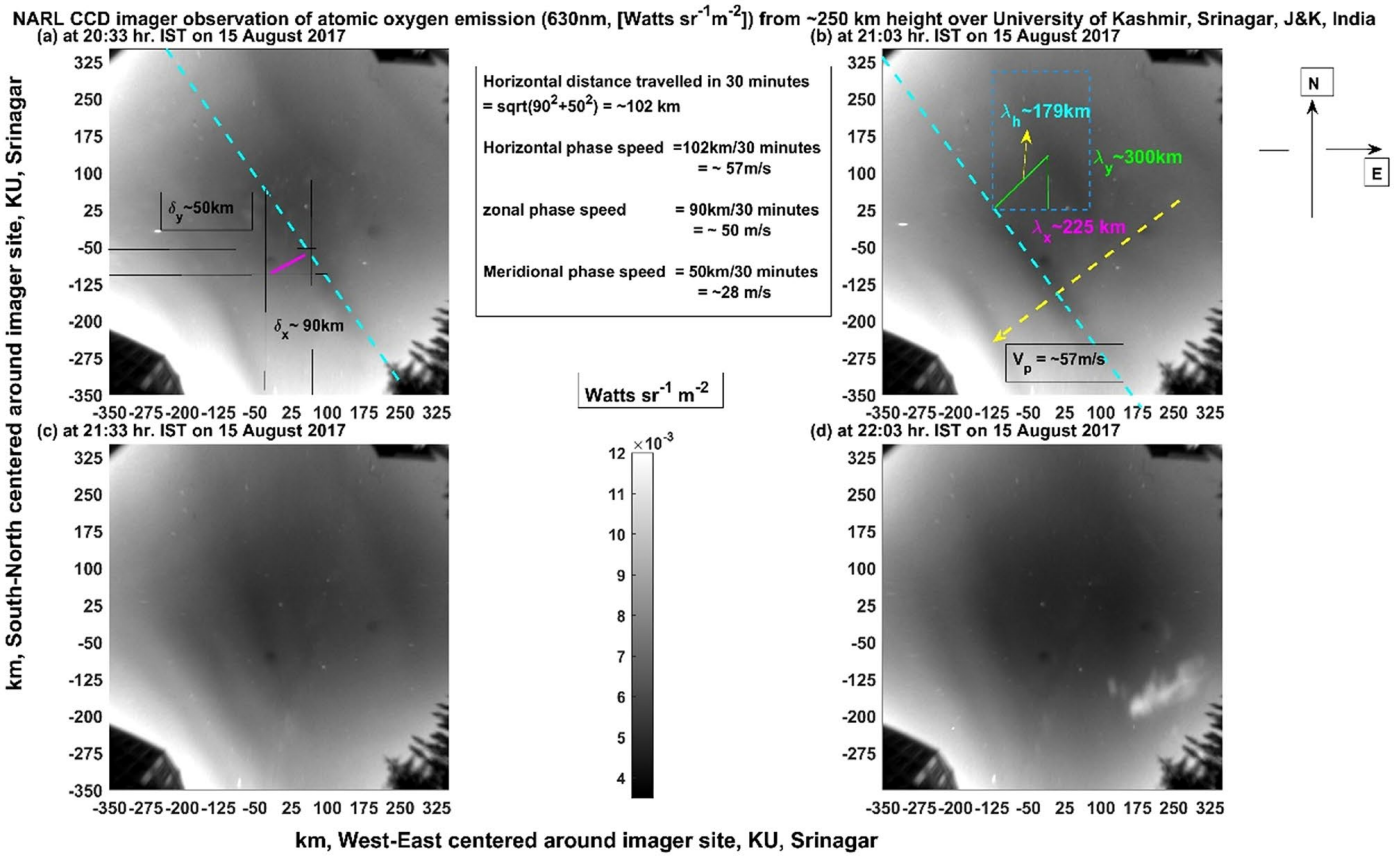
Illustrates the rectilinear-geographical spatial information of 630 nm emissions as shown in Fig. 1 (on 15 August 2017) for four scans separated by half an hour. While the y axes (zenith angle of − 57°–57°) run from South (bottom) to North (top), the x axes (zenith angle of − 57°–57°) run from West (left) to East (right) centred over KU, Srinagar. The colour bar values (emission intensity) are given in W sr^−1^ m^−2^ which can be converted into Rayleigh unit as 1 Rayleigh = 10^–9^/2πλ W sr^−1^ m^−2^ = 10^–3^/2π0.63 W sr^−1^ m^−2^ = 0.25 × 10^–3^ W sr^−1^ m^−2^.

Figure 2 shows un warped images of four panels of Fig. 1 in geographical coordinates (covering the pixel number ranges of 101:400 and 101:400 in the east–west and north–south directions respectively). They cover the horizontal space of ±  ~ 350 km in the north–south direction (y-axis) and in the east–west direction (x-axis) at the height of ~ 250 km over KU, Srinagar. This is in contrast to that of Fig. 1 which is in pixel coordinate (512 by 512). The image size is truncated to the pixels range 101–400 (instead of 1–512) which corresponds to maximum zenith angle (field of view) of ~ 57° for all the three filters to avoid unnecessary interfering signals (say surrounding street lights, buildings, trees) coming from nearby ground locations surrounding the imager site. Important quantitative information like (1) inter-band separation distance or horizontal wavelength, (2) band moving speed and direction and their time evolution is extracted from these images. It is also illustrated in Fig. 2 as to how the horizontal wavelength, speed and direction are estimated. It is observed that initially the horizontal wavelength (spatial separation between two white or dark bands) observed is about 200 km (~ ± 15 km, Fig. 2a) and within half an hour it became about 180 km (~ ± 10 km, Fig. 2b) and in the next half an hour it became about 60 km (± 8 km, Fig. 2c). In the next half an hour, the waves begin to disappear. As the bands cover the whole image, it is estimated that the total length of each of the bands is of the order of 900 km. Since the horizontal wavelength is significantly varying with time, it is difficult to find the exact speed of the moving phase front of these MSTIDs. The calculated speed of the phase front turns out to be around 57 m/s (~ ± 5 m/s). The four panels in this figure (Fig. 2) are separated in time by about half an hour beginning at 20:33 IST. The intensity values are obtained by taking in to account of the calibration of the imager at 630 nm but with band width of about 2 nm. The Van Rhijn and atmospheric extinction effects in the images are partially corrected by removing averaged signal strength from each of the pixels. Hence these effects do not influence the interpretation of the physical structure of the observed MSTIDs in any significant way. Removing them is, therefore, inconsequential.

**Figure 3:**
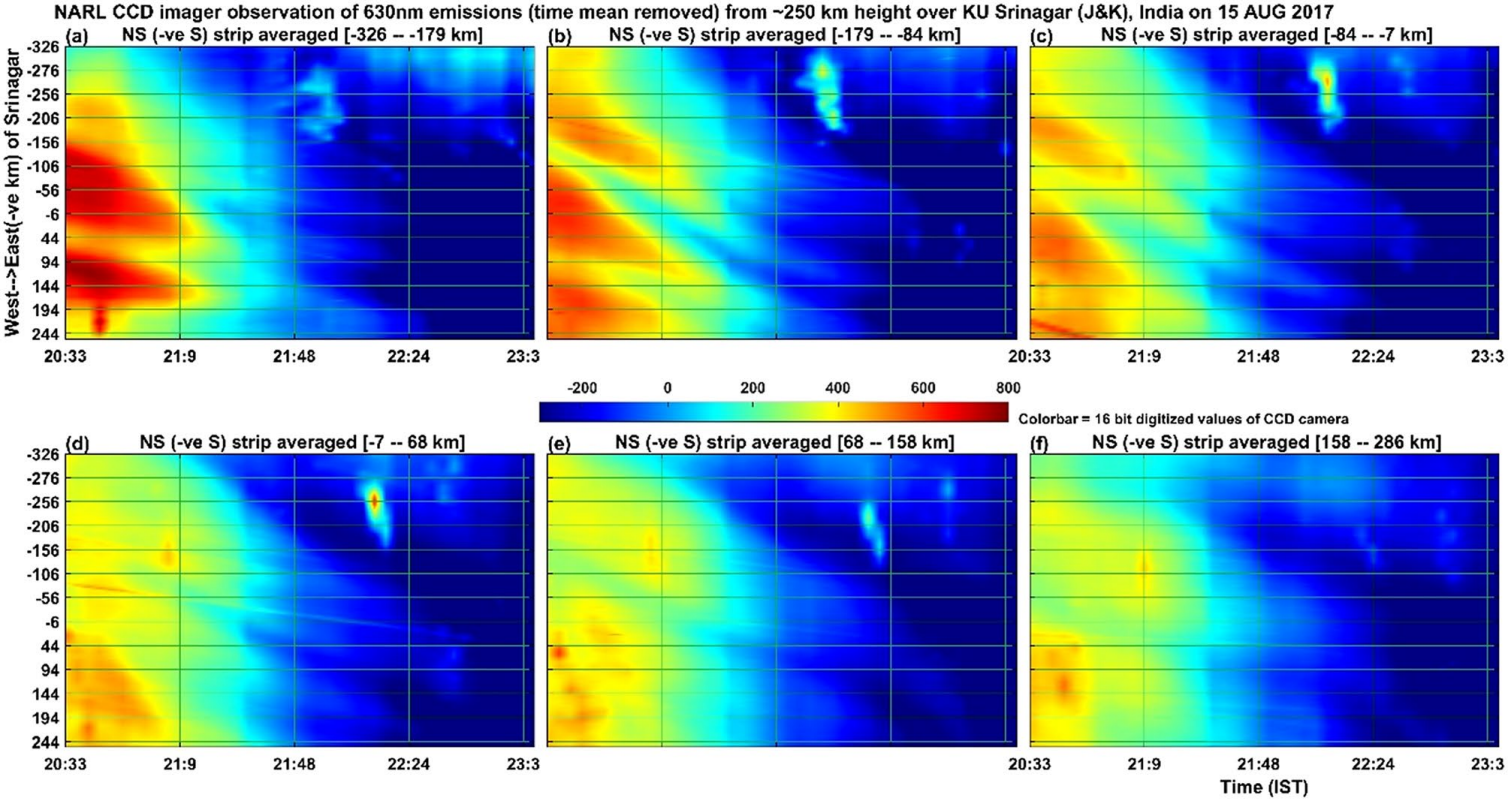
Depicts the keogram of 630 nm airglow images (as observed in the Fig. 1 on 15 August 2017) averaged in 50 bins in the North–South direction and their time evolution (x axes) is shown for the West (bottom) to East (top) direction in single pixel levels. The pixels are converted into rectilinear geographical coordinates for the height of 250 km centred over KU, Srinagar. −ve S in the title of images means negative values correspond to South of the imager location, which corresponds to the values mentioned within strip averaged**.**

Figure 3 illustrates the keogram images of 630 nm emissions at ~ 250 km height occurred on the night of 15 August 2017. While the x axes show the time period of observation, the y axes show the west–east evolution of north–south averaged 50 pixels. The horizontal north–south space covering 50 pixels is indicated on the top of the corresponding panels in which negative values denote the southern side of the imager location. Figure 3a shows the southern side averaged (179–326 km in the south side of the imager at 250 km height) intensity of the 630 nm emission and its time as well as east–west spatial evolution. It can be easily identified that in the first one hour (20:30–21:30 IST as observed in the Fig. 1), there is well-defined bright patterns of intensity moving towards the west. In Fig. 3b, covering the 84–179 km region in the South, it seems that there is an increase in the number of patterns in the east–west direction. The interesting thing to be observed here is that the intensity of these patterns gradually reduces in the northern side of the imager as noted in the bottom panels (Fig. 3). Also, it can be noted that the well-defined patterns of four bands in the southern (top panels of Fig. 3) side averaged intensity got diffused in the northern side (bottom panels of Fig. 3). The estimated zonal wavelength is ~ 225 km (± 15 km) and the phase front observed westward speed is about 50 m/s (~ ± 5 m/s). Actually, different bands show variable zonal phase speeds. Bands located in the far western side of the imager site shows westward phase speed of about 20 m/s (~ ± 4 m/s) but those located near the zenith of the site shows large phase speed of about 50 m/s (~ ± 5 m/s). Bands located in the far eastern side of the imager shows about 25 m/s (~ ± 6 m/s). Wave activities are seen until about 22:00 IST. Again, here, it can be noticed that the phase speed becomes slower with time and finally they moved mostly in the west direction with minimum speed. One more thing to be noticed here is that there are four bands visible in the first hour of observation (Fig. 3b). The bottom most band, located and aligned along the west side of the imager, remained almost stationary and all other bands move westward with increasing speed in the eastward direction (bright band). The band structures appear to move mostly westward and the band patterns are similar in the northern and southern most averaged plots. Further, after 21:10 IST, the bands intensities reduced with time and finally around 22:40 IST all bands disappeared.

**Figure 4:**
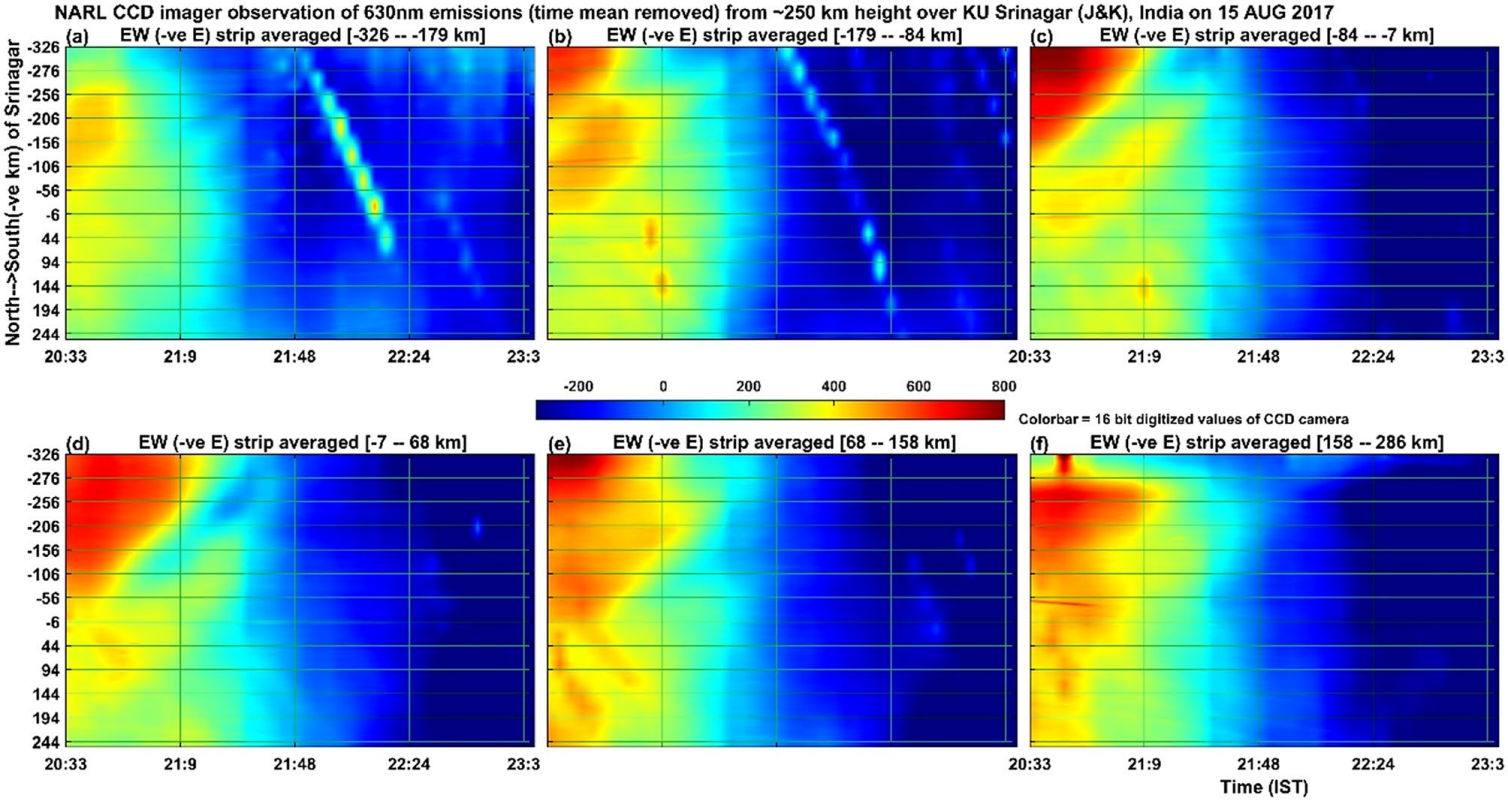
Shows the same as the Fig. 3 except that North–South and East–West are interchanged in the present keogram.

Figure 4 illustrates the same as Fig. 3 except that here north–south and east–west directions are interchanged. Almost in all the panels of Fig. 4, in the southern side of the imager (-ve y axis), the well-defined strong intensity bands move towards the south in the first one hour with southward phase speed of about 110 m/s (~ ± 10 m/s), meridional wavelength of ~ 200 km zonal wavelength of ~ 200 km and horizontal wavelength of about 142 km (~ ± 10 km). In addition to this, in the first hour, there are small scale bands which move towards the north in the northern side (+ ve y axes) of the imager. These features though weak extend for another one hour. The source mechanism of this northward moving small-scale bands might be due to the unstable ionospheric plasma conditions developed in the large-scale MSTIDS moving in the south-westward direction. Because the perturbed electric fields associated with MSTIDs and the earth’s magnetic field together can generate E × B drift of the plasma that flows orthogonal to the primary electric field of MSTIDs. In addition, there are beaded string (21:48–22:24 IST) types of intensities with signatures of movement in time from ~ 21:40 IST onwards, which are nothing but signals associated either with stars or cloud motions occurring in the lower atmosphere. In order to find whether the movement of observed band patterns towards the west or south is a regular phenomenon, the same analysis was carried out for the next day also i.e. August 16, 2017. No such wave pattern was observed on the August 16 night.

## Discussion and conclusions

From Fig. 1, one important observation is that after the dark and bright bands of 630 nm emission are aligned along the N-S direction, the bands lose their distinct shape and expand in the east–west direction and finally all the bands either disappear or merge into a single one marking the end of the life of TIDs or specifically MSTIDS as in the present case. It is to be recalled that while the Perkins instability makes the bands to align along the north-west and south-east directions, it is either the southward moving winds or winds associated with southward propagating atmospheric gravity waves that make these bands to move in the south-west ward direction in night times. While moving so, the interaction between the earth’s magnetic field and the time evolving perturbation electric field associated with MSTIDs causes E × B drift of the ionospheric plasma that in turn controls the time evolving plasma bands of MSTIDs. This would make both the inter band spacing and the band width, particularly the dark bands, to increase. Once these bands get aligned along the N-S direction, they come under the full control of the earth’s magnetic field. This may be one of the major reasons that the initial south-westward motion of MSTIDs finally becomes westward motion and then they dissipate. These mechanisms are well illustrated in all the panels of Fig. 1.

**Figure 5:**
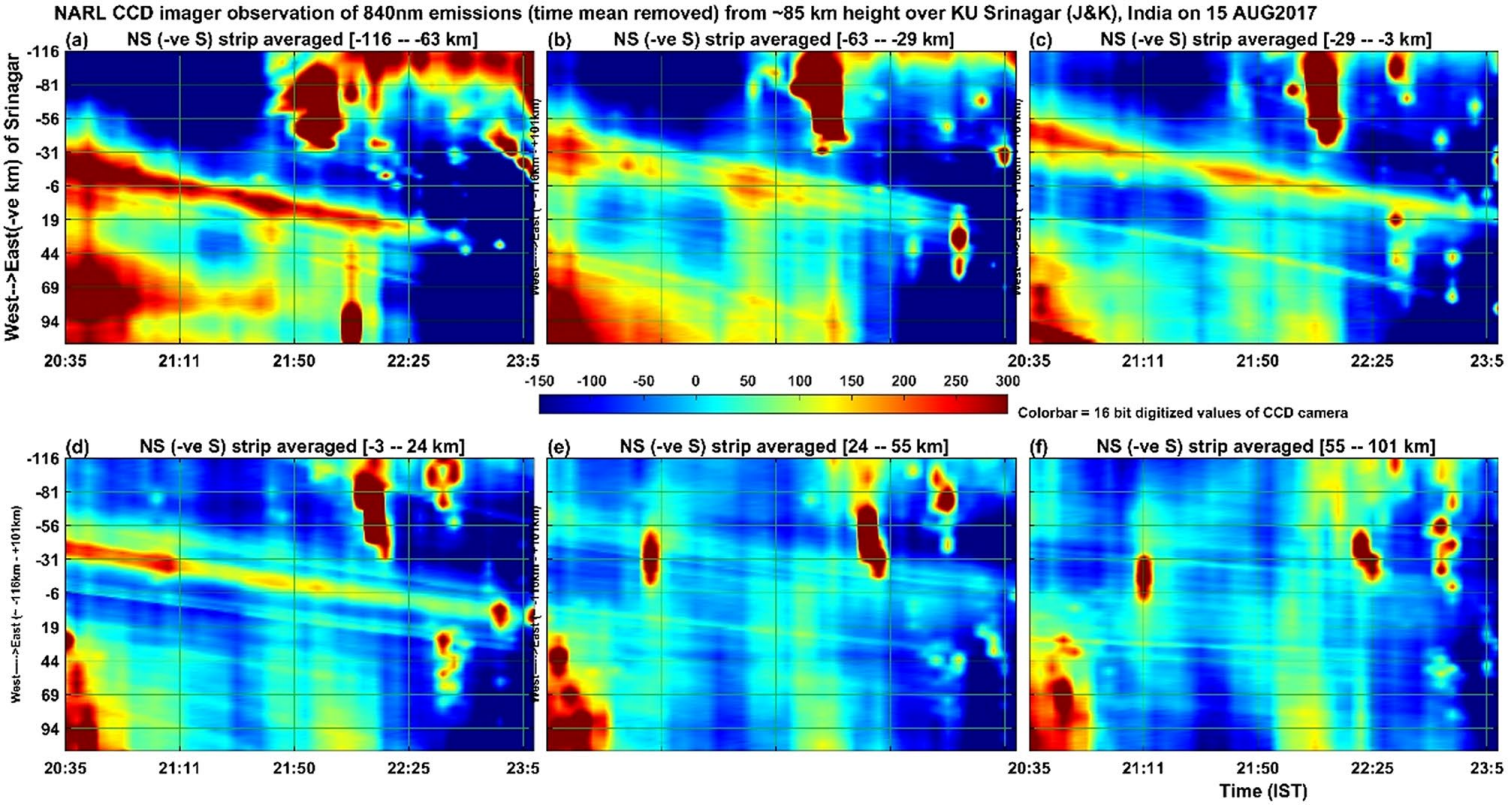
Illustrates the same as the Fig. 3 but for the 840 nm emissions of OH molecules from the height of about 86 km (maximum zenith angle covered is − 57°–57°). The colorbar values (emission intensity) are given in W sr^−1^ m^−2^ which can be converted into Rayleigh unit as 1 Rayleigh = 10^–9^/2πλ W sr^−1^ m^−2^ = 10^–3^/2π0.84 W sr^−1^ m^−2^ = 0.19 × 10^–3^ W sr^−1^ m^−2^.

**Figure 6:**
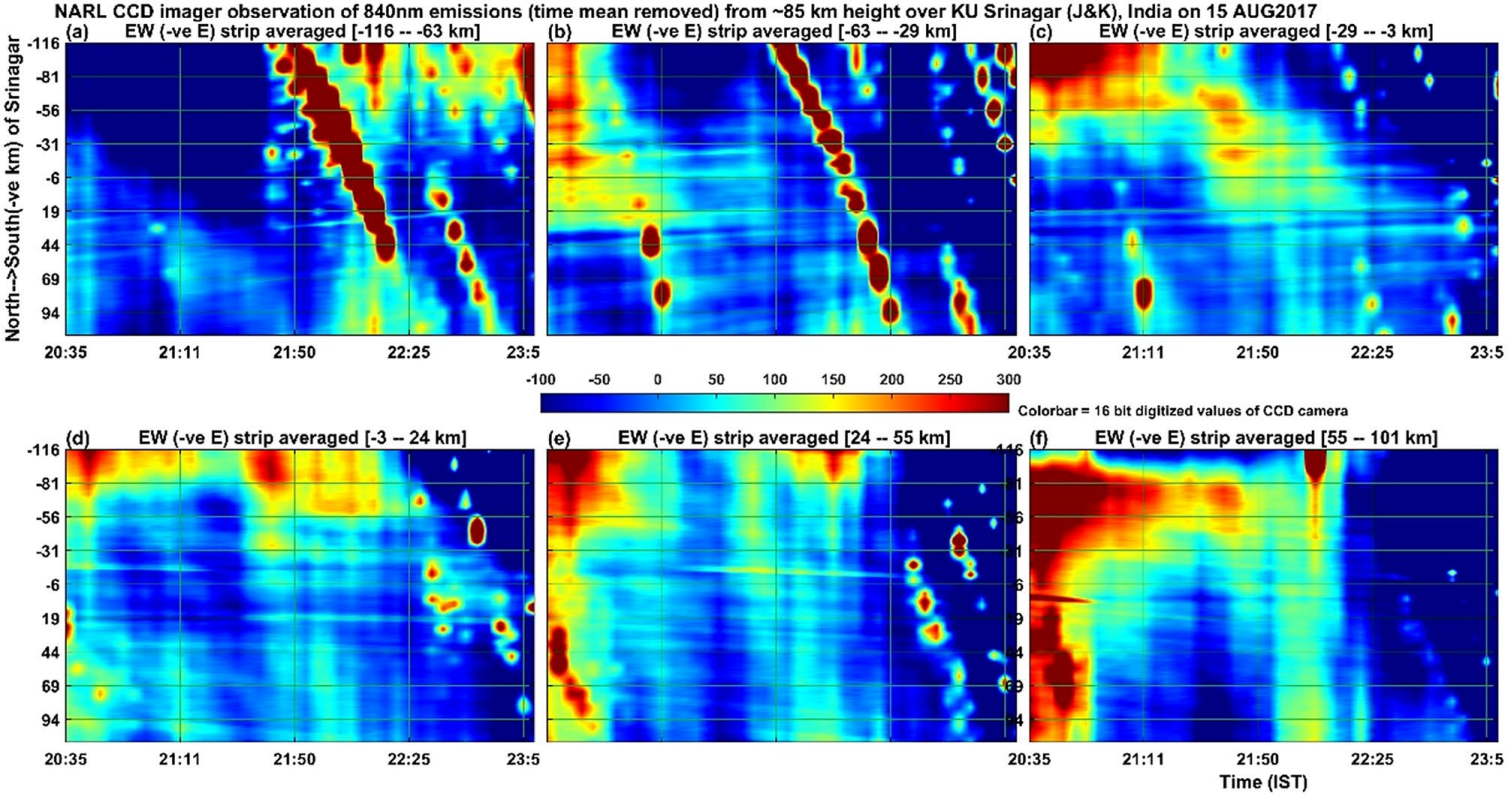
Illustrates the same as the Fig. 4 but for the 840 nm emissions of OH molecules from the height of about 86 km.

**Figure 7:**
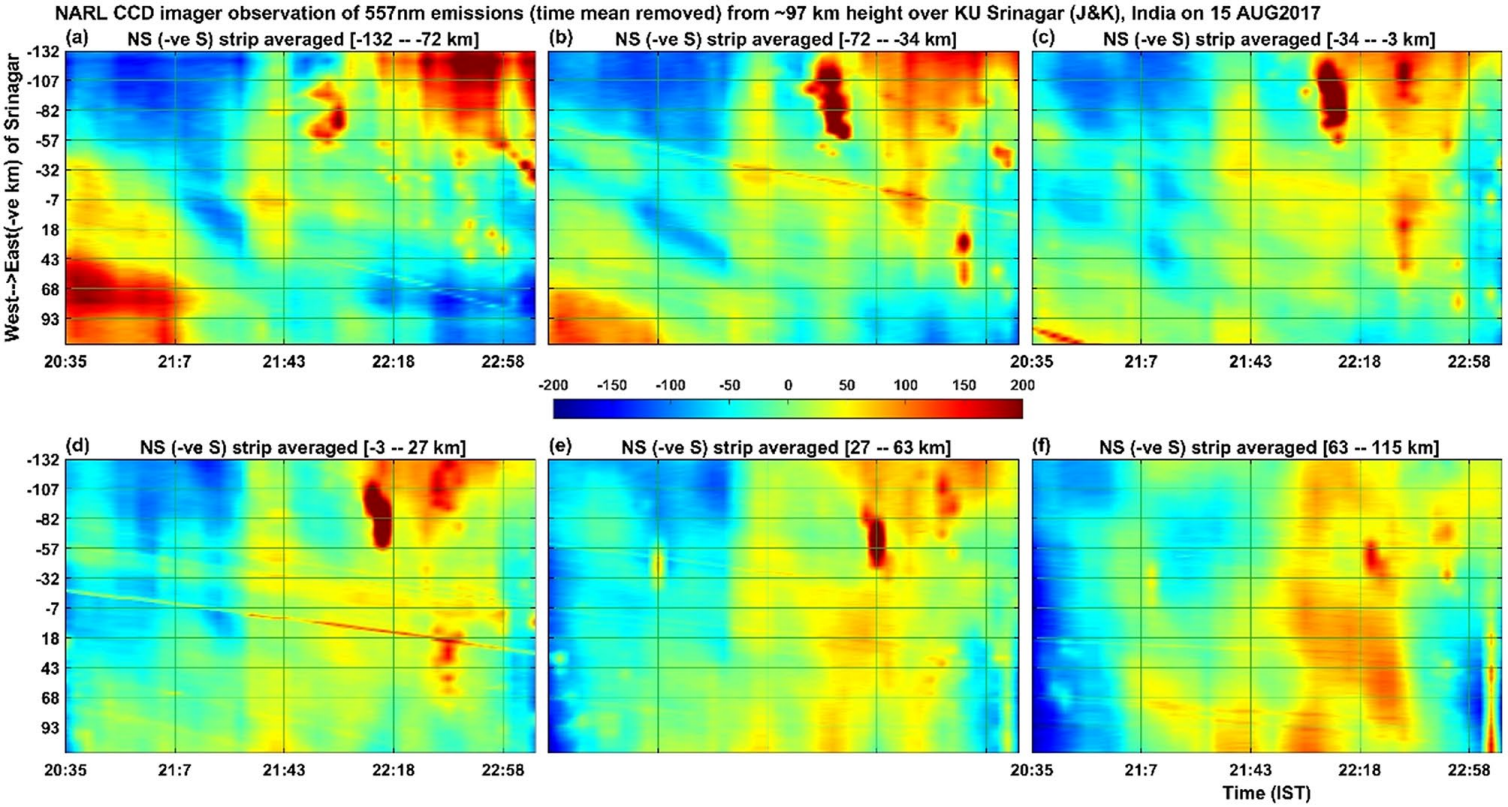
Illustrates the same as the Fig. 5 but for the 557.7 nm emissions of atomic oxygen from the height of about 97 km. The colour bar values (emission intensity) are given in W sr^−1^ m^−2^ which can be converted into Rayleigh unit as 1 Rayleigh = 10^–9^/2πλ W sr^−1^ m^−2^ = 10^–3^/2π0.557 W sr^−1^ m^−2^ = 0.29 × 10^–3^ W sr^−1^ m^−2^.

**Figure 8:**
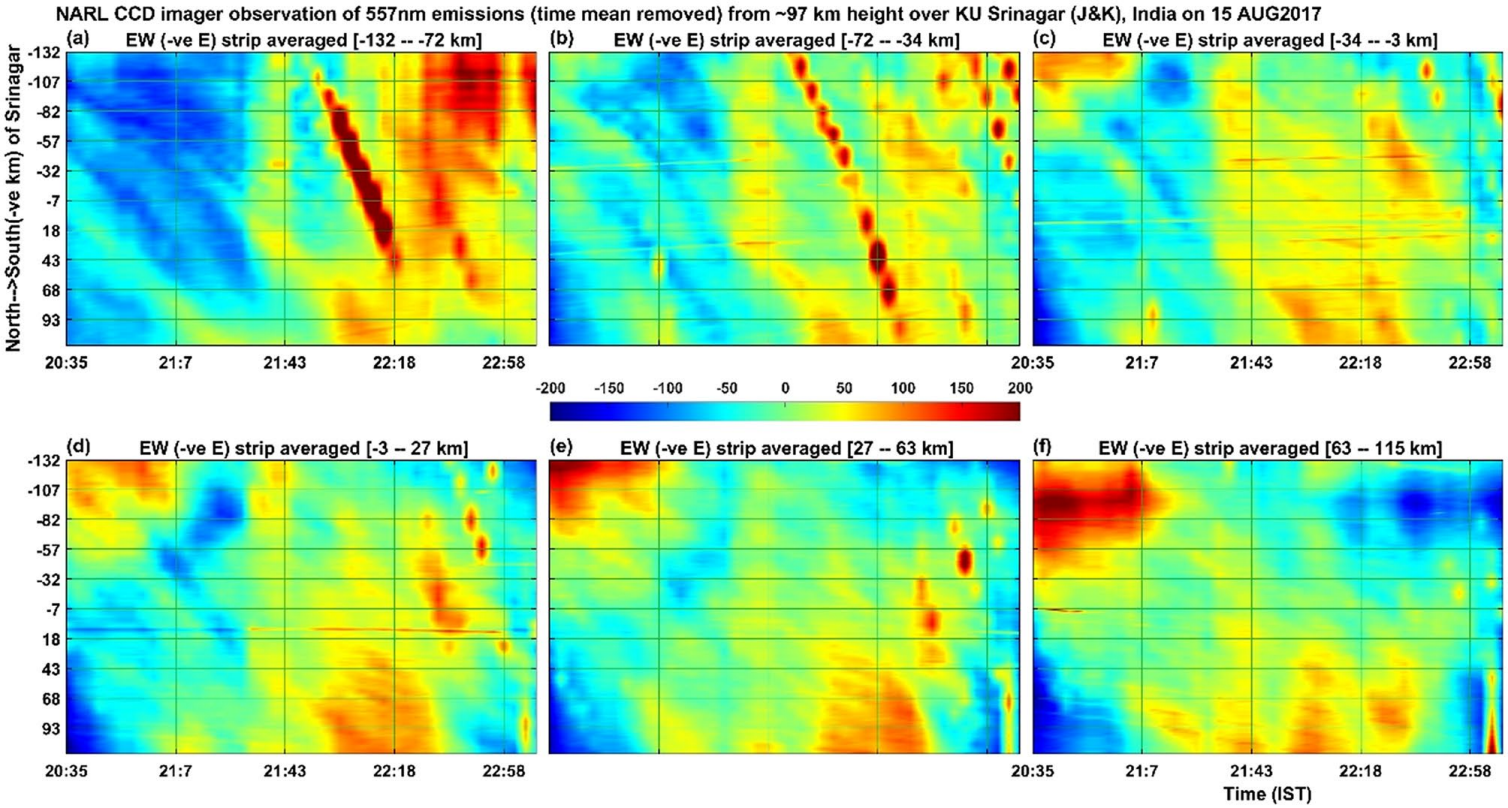
Illustrates the same as the Fig. 6 but for the 557.7 nm emissions of atomic oxygen from the height of about 97 km.

Now the source of the atmospheric gravity waves needs to be taken in to account. Figure 5 is identical to Fig. 3 except that it is now the airglow emissions at the centre wavelength of 840 nm associated with OH molecular emissions occurring at the height of about 85 km. It is interesting to note here that similar to Fig. 3 for atomic oxygen emissions at ~ 250 km height, OH emissions at 86 km (Fig. 5a) also show similar and distinct band patterns of bright emissions moving westward (~ 5 m/s) in the first one hour of observations. Figure 6 shows the same as in Fig. 5 except that it is now 50 bins averaged in the east–west direction and the features moving (~ 2.5 m/s) southward. The southward motion of bright patches of 840 nm emission intensity clearly indicates that the mesospheric gravity waves observed in the present work at the height of ~ 86 km propagated in the south-westward direction with moving speed of ~ 5.6 m/s. Figure 7 shows the same as Fig. 5 but for the 557.7 nm emission by atomic oxygen at the height of about 97 km. Similar to the Figs. 3 and 5, 7 (557 nm emissions in the height of ~ 97 km) also shows that there is a kind of band pattern moving westward in the first two hours in the southern side averaged (Fig. 7a–c) intensities. The northward moving band patterns visible in Fig. 8, which is for the 557.7 nm emission at ~ 97 km and is same as Fig. 6 except for the 840 nm emission, are thought to be due to in-situ generated gravity waves.

The simultaneous occurrence of bright and dark bands in all the three wavelengths (at the heights of ~ 85 km, ~ 97 km and ~ 250 km) in the first one hour of the present observation period would indicate that mesospheric gravity waves would have contributed to the appearance of MSTIDs in the F region ionospheric plasma. To our knowledge, this result is first of its kind showing simultaneous occurrence of west ward moving airglow band at the heights ranging from 85 km to about 250 km. The present observation of northward (Fig. 8) moving patterns of 557.7 nm emission at ~ 97 km, southward (Fig. 6) moving patterns (many bands) of 840 nm emission at ~ 85 km and the south ward (Fig. 3) moving ionospheric plasma at ~ 250 km (only a few bands associated with MSTIDs) raises an important question that how the MLT region (mesosphere and lower thermosphere) small horizontal wavelength atmospheric gravity waves would have led to generation of large horizontal wavelength MSTIDs in the F-region ionosphere. Further, Perkins instability mechanism demands southward moving winds generate south-westward propagating MSTIDs (Jonah et al. 2016). The southward moving atmospheric gravity waves in the mesosphere can lead to the generation of MSTIDs. To explain this, we calculated the vertical wavelength of the gravity waves observed in the 840 nm emission (~ 85 km height) by using the gravity wave dispersion relation Hines^43^

1 $$ {\text{m}}^{2} = {\text{N}}^{2} /\left( {{\text{U}} - {\text{c}}} \right)^{2} - {\text{k}}^{2} {-}1/4{\text{H}}^{2} $$

where m = 2π/λ_z_ is the vertical wave number of gravity waves.

N = 2π/T_B_ is the Brunt–Vaisala (BV) frequency and T_B_ is the BV period ~ 6 min in the mesosphere region near 85 km.

The BV period T can also be derived using the relation

$${\text{T}}_{{\text{B}}} = 2\pi /\sqrt {\left[ {\left( {{\text{g}}/{\text{T}}} \right) \times \left( \Gamma{_{{\text{a}}} -\Gamma_  {{\text{e}}} } \right)} \right]}$$

where g is the earth’s acceleration due to gravity, T is the atmospheric temperature (Kelvin), Г_a_ is the atmospheric dry adiabatic lapse rate and Г_e_ is the environmental lapse rate.

k = horizontal wavenumber; H is the atmospheric temperature scale height ~ 8 km in the mesosphere.

In the present work, it is obtained ~ 25 km and ~ 15 km as zonal wavelength (0.04 per km wavenumber) and meridional wavelength (0.06 per km wavenumber) respectively. The horizontal wavenumber is = sqrt[(0.04*0.04) + (0.06*0.06)] = sqrt[0.0016 + 0.0036] = sqrt(0.0052) = 0.07; horizontal wavelength = 1/0.07 = 14.2 km.

Here c, phase speed of the gravity waves and k can be obtained from the imager data of OH emissions in the height of ~ 85 km.

The values obtained from the analyses of the images of the present work are:

c = − 5 m/s (present OH image data)

k = 2π/14.2 km (present OH image data)

**Figure 9:**
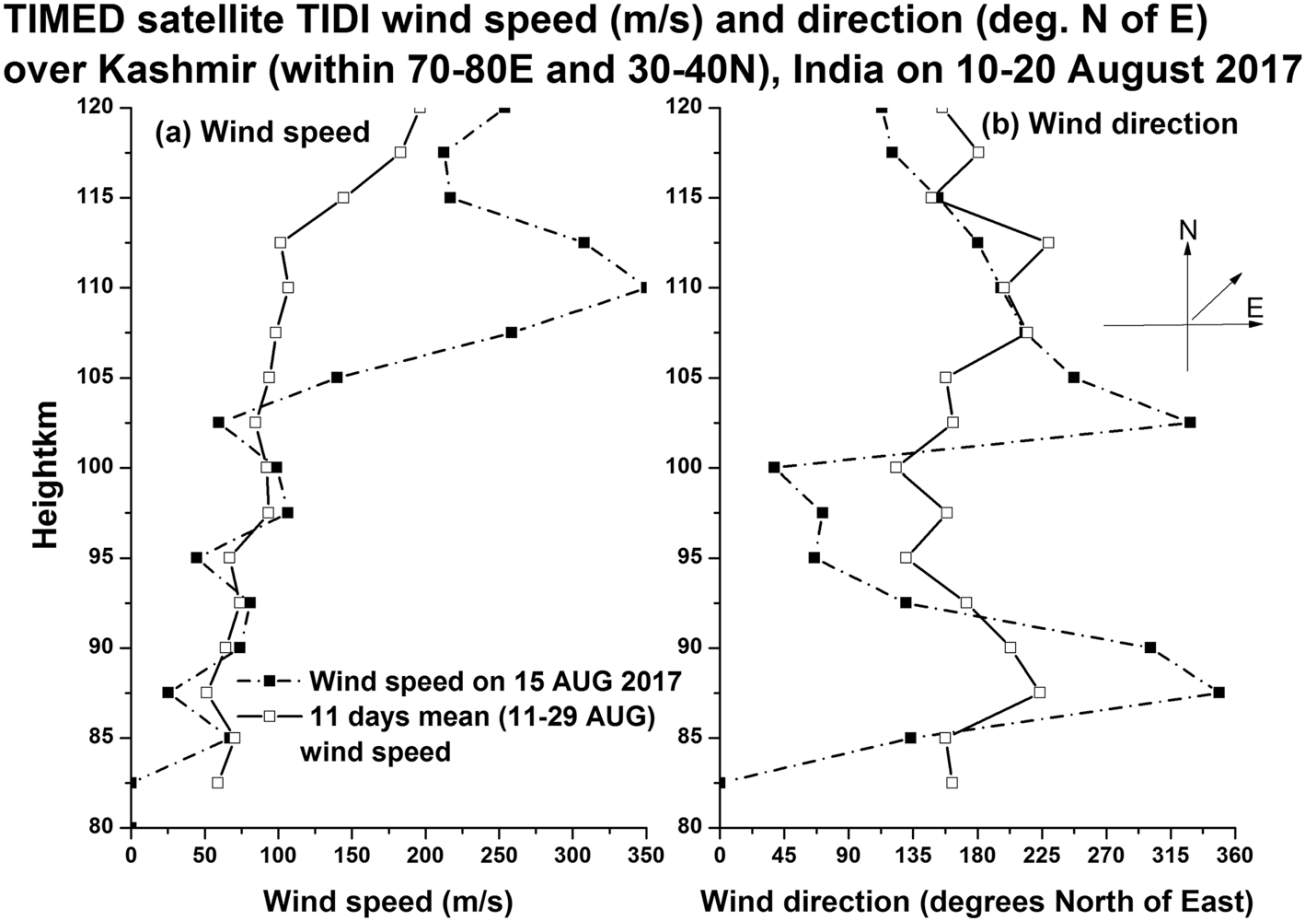
Provides the height profiles of horizontal wind speeds (m/s, TIDI-TIMED satellite data) at 12.6 UT on 15 August 2017 and on 10–20 August 2017 (11 days mean wind) for the longitude and latitude centred (± 5°) around the CCD imager site in Kashmir, India.

U = − 75 m/s (TIDI (TIMED satellite data for the height of ~ 85 km, Fig. 9a, b)

In the Fig. 9a (height profile of horizontal wind speed), it can be noticed that the TIDI horizontal wind speed at 85 km height is ~ 75 m/s in both the cases of 15 August 2017 and 11 days (10–20 August 2017) mean wind (background wind). In the Fig. 9b (height profile of horizontal wind direction), the direction of the horizontal wind is ~ 135 degrees north of east on 15 August 2017 and ~ 156 degrees north of east for the 11 day mean wind (background wind), which makes the wind value of ~ − 75 m/s at ~ 85 km height.

With these values inserted in Eq. (1), the value of the square of the vertical wavenumber (m^2^) becomes negative and hence these waves can be classified as external or transient gravity waves that cannot propagate above this height of ~ 85 km. Similarly, the gravity waves at ~ 97 km height associated with 557.7 nm emission also shows negative values for m^2^ and hence this wave also cannot propagate to higher heights and get dissipated there itself. It is interesting to note here that at the height of ~ 97 km (Figs. 7 and 8, atomic oxygen emission, 557.7 nm), the southward propagating gravity waves observed in the heights of ~ 85 km (OH emission; 840 nm) disappeared but there is a presence of northward propagating waves. It is interesting to understand what would have happened to the mesospheric (~ 85 km) gravity waves in the lower thermosphere (~ 97 km) and how other new gravity waves are generated there at ~ 97 km height? Fig. 9b indicates that at ~ 85 km height the horizontal wind was westward and at ~ 87 km height it became eastward and again it became westward at about 92 km height. It is speculated here that the strong wind shears in the heights of 85–92 km would have led to the dissipation of the gravity waves observed in the mesospheric heights of ~ 85 km. The observed northward moving gravity waves at ~ 97 km height (Fig. 8) is possibly due to in-situ generated secondary gravity waves that arose due to either dissipation of the upward propagating mesospheric gravity waves or any other tides or planetary waves or a mixed of all these waves. It is taken as a future exercise to determine the exact source mechanism of the northward propagating gravity waves in the height of ~ 97 km during the first few hours of observation on 15 August 2017. It seems that the secondary gravity waves observed in the height of ~ 97 km would have been dissipated in the higher heights of about 107 km (Fig. 9b) as there is another strong vertical shear (changing from eastward to westward) of horizontal wind near this height (reversal of wind near 107 km in Fig. 9).

**Figure 10:**
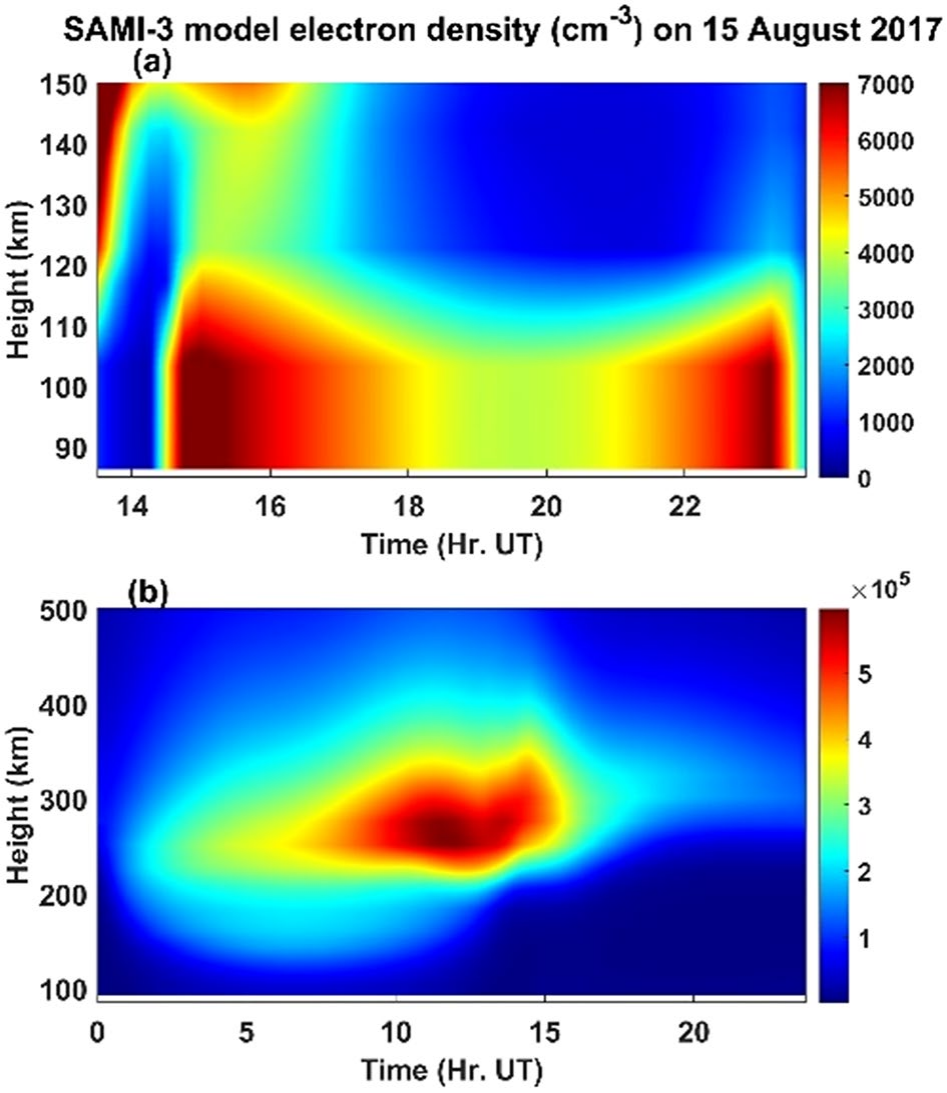
Presents the results of SAMI3 ionosphere model (**a**) time evolution (13:30 – 24:00 UT) of electron density in the heights of 85–150 km for the Srinagar location (75° E; 34° N geographic) on 15 August 2017 (**b**) same as a except for the heights of 85–500 km and time of 00:00–24:00 UT. 1.3 and 8.3 km are the height resolutions for (**a**) and (**b**) respectively.

**Figure 11:**
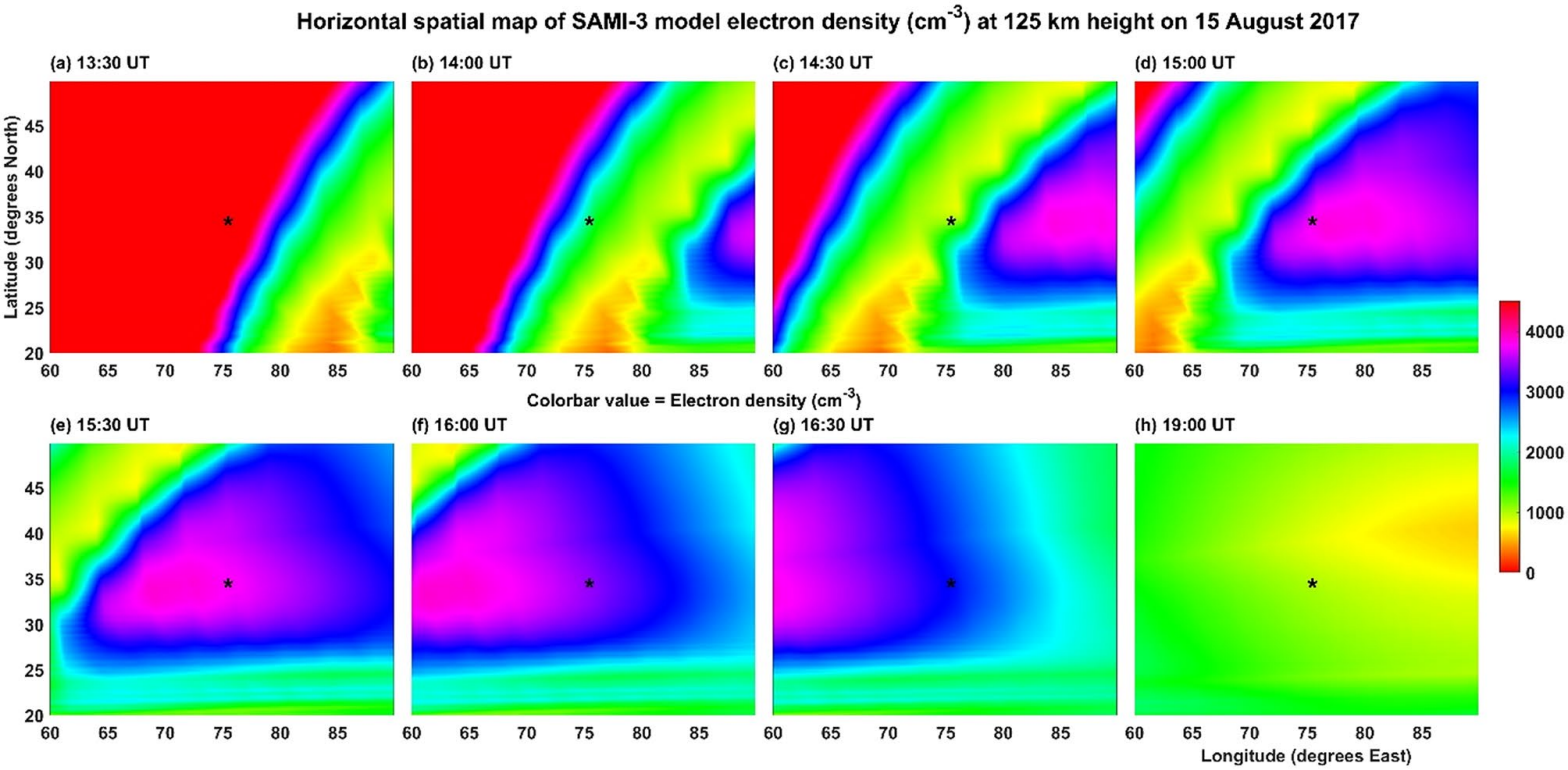
Ilustrates the contour plots (x and y axes for longitude and latitude respectively) of electron density (SAMI3 model) at the height of 125 km centred over the CCD imager location of Srinagar, at (**a**) 13:30 UT (**b**) 14:00 UT (**c**) 14:30 UT (**d**) 15:00 UT (**e**) 15:30 UT (**f**) 16:00 UT (**g**) 16:30 UT and (**h**) 19:00 UT. 0.15° is the resolution in latitudes and longitudes. The CCD imager location in Srinagar is marked as star symbol at the geographical longitude of 75°E and latitude of 34°N.

Given this scenario of observation of gravity waves near the height of ~ 85 km (OH emission, 840 nm, Figs. 5 and 6) and their dissipation in the higher heights of about 92 km and the generation of secondary gravity waves at this height along with their possible dissipation around 107 km, how could atmospheric gravity waves would have contributed to the generation of MSTIDS in the present study. This is an intriguing question that demands convincing answers to explain the exact generation mechanisms of MSTIDS. Here it is needed the knowledge of basic physical principles of generation of both the MSTIDs and the contribution of atmospheric gravity waves to them for their growth in the F region ionosphere. Recently, Chum and Podolska^21^ reported that 3D analysis of GWs in the F region ionosphere could confirm their association with lower atmospheric sources. Full physics- based model simulations of generation and dissipation of atmospheric gravity waves and their coupling with ionospheric plasma physics can shed light on this issue. Figure 10a shows the SAMI3 ionosphere model results of height profiles (85–150 km) of electron density and their time evolution (13:30 – 24:00 UT; 19:00 – 05:30 IST on 15 August 2017) over the CCD imager site of Srinagar. It can be observed that above the height of 85 km and up to the height of about 110 km, the electron density varies at high frequencies (time) comparing to those at heights higher than 110 km. This would indicate that direct influences of mesospheric and lower thermospheric gravity waves on the ionospheric plasma would have been limited to heights below 110 km. Above this height, secondary gravity waves generated through dissipation of upward propagating lower atmospheric gravity waves could be one of important source mechanisms of periodic motions in ionospheric plasma irregularities. The influences of these secondary waves on ionospheric plasma can be seen even up to above the peak (electron density) of the F region ionosphere. Figure 10b illustrates this phenomenon clearly; here one can see the influence up to the height of ~ 500 km. Figure 11 illustrates the time evolution of horizontal map of (60°E–90°E; 20°N–50°N; geographic) SAMI3 model electron density at the height of 125 km where the signature of MSTIDs started to be visible from below. As the centre of this figure approximately matches the location of the CCD imager site, Srinagar (75°E, 34°N; geographic), it can be seen clearly how the periodic variation of electron density portrayed. At 13:30 UT (19:00 IST, Fig. 11a), there is no clear periodic variation of electron density noticed except for its gradual decrease with longitude which is normal and expected as our earth rotates and hence decreasing ionizing radiation from sun for the E region ionosphere. Oscillating patterns started to be visible at 14:30 UT (Fig. 11c) and at 15:00 UT (Fig. 11d) it is clearly seen the oscillations in electron density in the latitude region of ~ 37°–45° N. The oscillating patterns, drifting mostly westward with time, is disappeared at 16:30 UT (22:00 IST, Fig. 11g) in regions beyond 67°E. The present imager data also showed similar time variations of MSTID (Fig. 1). This shows that the SAMI3 model finely reproduced the MSTID structures as observed by the CCD imager over Srinagar.

It may be noted that the structures of MSTIDs in SAMI3 model are not similar to what imager observed in the present work. Here we point out that we carried out only normal SAMI3 model. To clearly see the unambiguous MSTIDS in SAMI3, we need to introduce perturbations in the atmospheric winds and temperature associated with breaking gravity waves in the SAMI 3 numerical simulation model. Duly^52^ showed MSTIDS generated in the SAMI3 model by introducing random perturbations in one case and in another case by introducing specific gravity wave k vector. In the latter case, finally MSTIDS are generated through mechanisms associated with earlier case. This shows that perturbations associated with dissipating gravity waves act like seeds for the generation of MSTIDs which are highly electrodynamical in nature as per the Perkins instability theory of MSTIDs. In the present work, atmospheric gravity waves observed at ~ 85 km and ~ 97 km are highly dissipative in nature because of the nature of background atmospheric winds. It is to be noted that SAMI3 model is yet to develop fully to account for the sporadic E irregularities without which it is impossible for the model to determine the exact propagation direction of MSTIDs^52^.

### Secondary gravity waves leading to generation of MSTIDs

As the mesospheric and lower thermospheric gravity waves seen in the heights of ~ 85 km and ~ 97 km would have possibly dissipated below ~ 107 km because of the strong wind shears at the heights of ~ 90 km and ~ 105 km, it is imperative to find a logical reason to link these observed waves with simultaneous appearance of MSTIDs over the CCD imager site of Srinagar. As an interesting work supporting the present observations, Vadas and Liu^8^ reported the generation of fast- and large-scale secondary gravity waves in the lower thermosphere from the dissipation of upward propagating lower atmospheric convectively-generated slow-moving small and medium scale gravity waves. Using the high-resolution NCAR Thermosphere-Ionosphere-Mesosphere-Electrodynamics General Circulation Model (TIME-GCM) they studied the response of the thermosphere and ionosphere to the dissipation of the gravity waves generated from a single deep convection in the lower atmosphere. They successfully simulated the generation of large scale travelling ionospheric disturbances (LSTIDs) due to the propagation of the excited, large-scale, secondary GWs with horizontal wavelengths of the order of more than 2000 km in the heights of 120–250 km. The convectively generated gravity waves had horizontal wavelength of ~ 40–150 km, vertical wavelength of 50–65 km, and period of 10–20 min and their dissipation in the lower thermosphere led to the generation of secondary gravity waves with horizontal wavelength of the order of 2000 km, period of 80 min and horizontal speed of 480–510 m/s. Similar further studies^44–46^ also reported excitation of secondary gravity waves with horizontal wavelengths of ~ 100–4000 km and phase speeds of ~ 100–500 m/s in the heights of 120–250 km due to dissipation of upward propagating gravity waves. It is also claimed that these secondary large- scale gravity waves can propagate globally and vertically beyond above 400 km and induce moving ionospheric plasma disturbances like MSTIDs or LSTIDs^46,47^.

For the present report, it can be argued that the simultaneous occurrence of MSTIDs in the ionosphere and the gravity waves in the mesosphere (OH, 85 km) and lower thermosphere (O, 97 km) can be completely independent of each other. To strengthen the point of view that they are strongly linked to each together, one can compare the time period and evolution of the mesospheric gravity waves (Fig. 5) and MSTIDs (Fig. 3). It is outstandingly matching and both the signals lose their significance after 22:00 IST. Then the next question to be addressed here is that when gravity waves take hours to reach the ionosphere from the mesosphere, how can they simultaneously be present. It is to be noted here that the south-west ward propagating gravity waves detected at ~ 85 km are evanescent ones (m^2^ is negative at ~ 85 km height where the waves were detected in the imager as mentioned above) and the strong wind shear in the heights of 85–95 km (Fig. 9) would have dissipated them there. The turbulence generated thus would have generated secondary gravity waves above about 100 km and these secondary gravity waves would have influenced the E region electric field leading to the appearance of MSTIDs in the SAMI 3 model (Figs. 10 and 11). At around 22:30 IST, the simultaneous disappearance of MSTIDs in the 630 nm emission (Fig. 3) and in the SAMI3 electron density (Fig. 11g) and gravity waves in the mesosphere (Fig. 5) over the Srinagar site (75°E) clearly illustrates that they are strongly related to each other. Furthermore, if the observed MSTIDs were generated somewhere in the mid latitudes and they just travelled over Srinagar, then they should be still travelling and visible in the SAMI 3 model (Fig. 11g). But they disappeared in all the observations simultaneously.

Another interesting feature is the observation of a secondary generation of ionospheric plasma motions that move in the northward direction (Fig. 4) in contrast to the MSTID motion which is in the southward direction. The more interesting characteristic of the secondary plasma motion is that it is restricted to be only generated in the northern side of the imager site. A further detailed analysis of this observation will surely lead to more understanding of the characteristics of locally generated MSTIDs, which is also taken as the future scope of the present study.

### Western Himalayan topography link with mesospheric gravity waves

**Figure 12:**
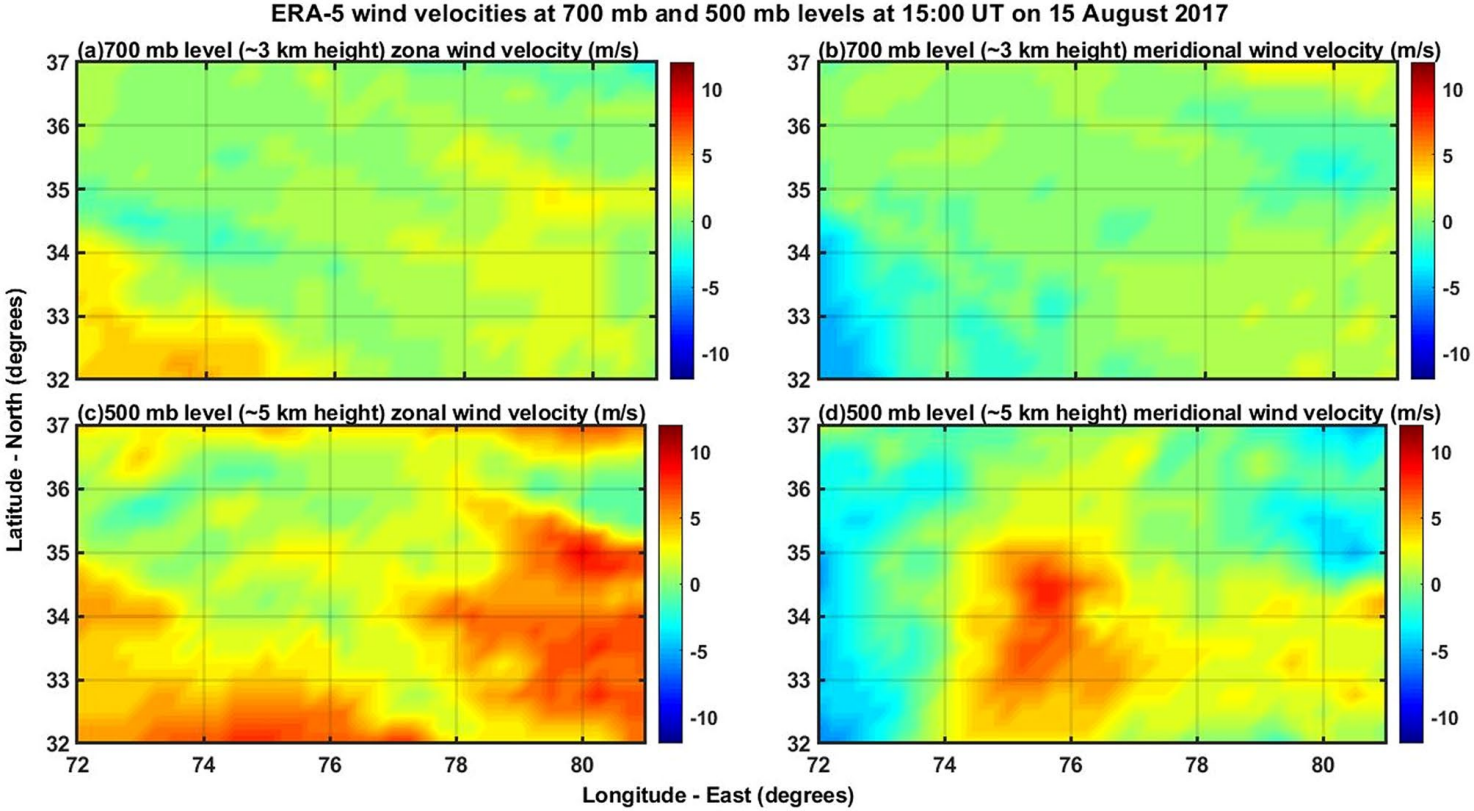
Depicts the ECMWF reanalyses 5 (ERA-5) zonal and meridional wind velocities at 700 mb and 500 mb levels at 15:00 UT on 15 August 2017.

**Figure 13:**
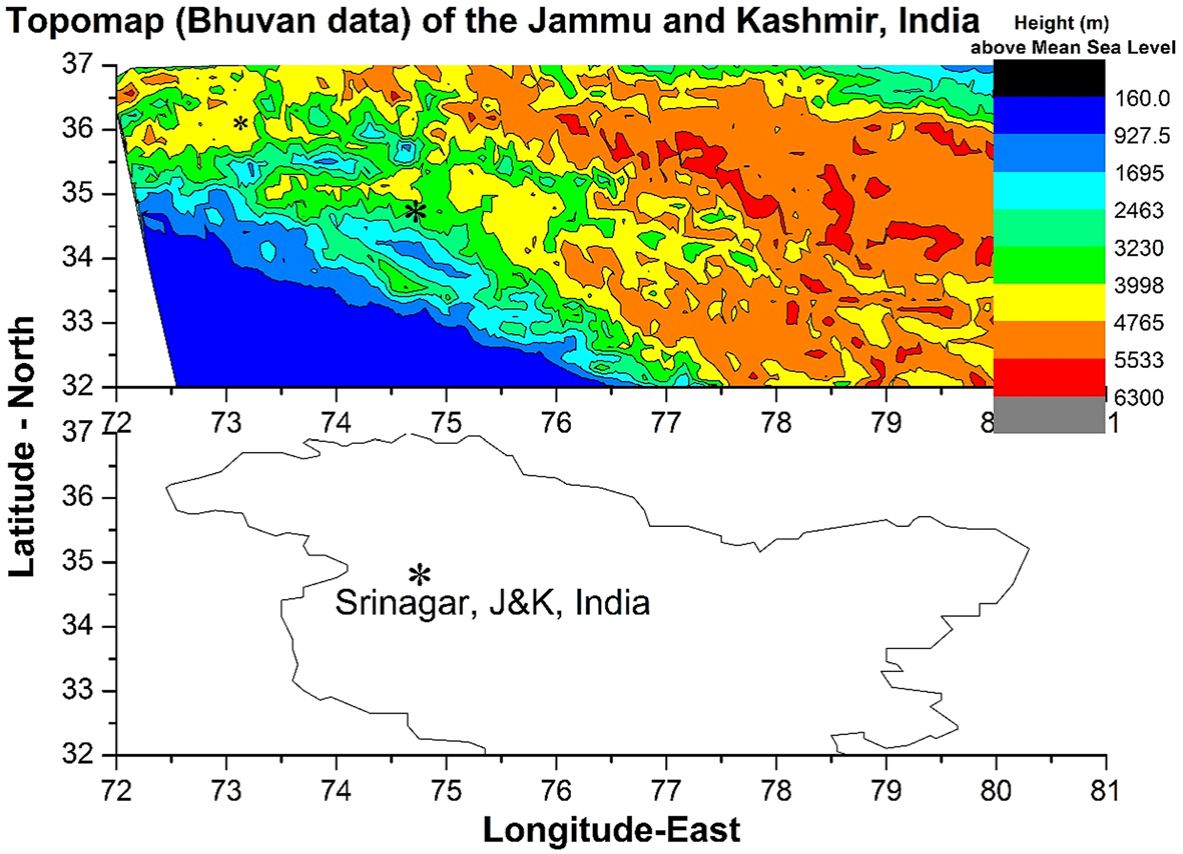
Shows the topographic (ISRO-Bhuvan data) structure of the erstwhile state of Jammu and Kashmir (J&K), India corresponding to the Fig. 10. The imager station, Srinagar, marked as asterisk symbol, is located in the present J&K Union Territory of India. The outline of the map is obtained from digital values of latitudes and longitudes associated with geographic outline of India as available in the official Indian government portal website of https://indiamaps.gov.in/soiapp/.

Since the filter with 840 nm as central frequency is actually a wide band (~ 720–920 nm with blocking notch at ~ 866 nm) filter in the near infra-red (NIR) region, its scan time is only ten seconds otherwise the receiver CCD camera would be saturated. As the lower atmospheric cloud patches can emit strong NIR signals, it is possible to determine their horizontal velocities by assuming that they are located at around 5 km height. Exact height determination of cloud patch heights is possible by triangulation method but it needs a minimum of three imagers separated in location by tens of kilometres distance. With single imager there may be error in the assumption of 5 km height of the clouds but the determination of speed of clouds will not change drastically as the clouds are normally spread within few kilometres of height. So, it is reasonable to assume that the cloud patches observed in the 840 nm filter are at 5 km height. As a result, the signals obtained using this filter will contain both the OH emission from the height of ~ 86 km as well as the emissions from cloud patches in the lower atmosphere. In Fig. 6, for the keogram of time evolution of north–south spread of emissions of 840 nm averaged in the east–west direction, it can be easily identified that there are many series of small blobs of high intensities moving towards the north direction with more intensity in the eastern side (Fig. 6a–c) of the imager. By assuming that the cloud patches are located at the height of about 5 km, the northward speed of the clouds is estimated to be about 12 m/s (~ ± 2 m/s), which is obtained from the rough measurement of time taken by a single cloud patch (thick blob of high intensity) to move from the south to north end as in the Fig. 6b. This is in excellent agreement with ERA-5 reanalyses meridional wind velocity at 5 km (Fig. 12d) over the Kashmir region. Figure 13 shows the topography of the Kashmir region corresponding to Fig. 12. It seems that northward moving winds (Fig. 12d) in the lower atmosphere would have generated the lower atmospheric gravity waves by hitting over the Himalayan mountain range from the south. Wind disturbances created in the upwind streamside because of the wind flow from the south to the north would have converted into southward propagating gravity waves in the higher heights and propagated vertically up into the mesosphere and lower thermosphere region over Srinagar (upwind streamside valley region). This may be one of the reasons that MSTIDs are more intense (Fig. 3a–c) in the southern side of the imager rather than in the northern side (Fig. 3d–f) in the present work. This would lead to the speculation that the MSTIDs reported in the present work were not generated in the mid latitude region but they would have occurred as a result of lower atmospheric gravity waves generated due to the effect of wind-flows over topography of mountains of the western Himalayas in the Kashmir region. The observed gravity wave patterns in the mesospheric heights (840 nm, Fig. 5) of about 85 km further supports our inference that it is the locally generated atmospheric gravity waves that would have led to the generation of MSTIDs over the Kashmir region.

Normally, it is believed that topography generated gravity waves will have zero phase speeds but the present observed mesospheric gravity waves possess phase speeds of about − 35 m/s (± 5 m/s). Then question arises as to how the high phase speed gravity waves observed in the mesosphere can be associated with topography in the lower atmosphere. If wind flows over mountains generate non-hydrostatic gravity waves then they will have phase speeds depending on the wave generation mechanisms and the background atmospheric wind and temperature conditions in which the waves are generated and propagated^48^. If hydrostatic gravity waves are generated then they will have stationary phase speeds. Here we don’t know what kind of gravity waves were generated because of wind flows over the Himalayas in the lower troposphere. Determining the gravity waves characteristics generated in the lower atmosphere needs lot of atmospheric wind and temperature information surrounding the Himalayas as well as in the higher levels of the atmosphere where the waves are propagating vertically upwards. As we don’t have observational information regarding these physical processes, it is speculated that the gravity waves generated there would have non-hydrostatic character and acquired high speeds in the mesosphere. Model simulations can help but that is beyond the scope of the present work and it is taken as future scope. However, the basic question here is that whether this wind speed of ~ 12 m/s at ~ 5 km height near the CCD imager site of Srinagar is sufficient for the generation of atmospheric gravity waves in the troposphere. Durran^8^ showed how this parameter, NH/U where N is the Brunt-Vaisala frequency, H is the height of mountain and U is the background wind speed, can shed light on whether gravity waves can be generated with the given wind velocity and mountain height.

For a bell-shaped mountain (with height H km) in the troposphere,

N = 1/B_T_ = 1/(5 × 60) = 1/300 = 0.0033 (typical in the troposphere)

where B_T_ is the Brunt-Vaisala period.

H =  ~ 4 km = 4000 m (Fig. 13)

U =  ~ 12 m/s (Fig. 12)

NH/U = 0.0033 × 4000/12 = 1.11

According to Durran^49^, this value of NH/U = 1.11 can lead easily to generate vertically and horizontally propagating gravity waves and hence it can be said that the lower atmospheric topography and wind conditions near the CCD imager site of Srinagar were well suited for the generation of atmospheric gravity waves that can propagate vertically as well as horizontally up into the mesosphere. It is to be reminded here that not only topography generated gravity waves can propagate to ionosphere but other mechanisms also can lead to generation and propagation of GWs to F region heights from below; namely, waves leaking from wave guide formed by wind shear and temperature profile around mesopause region^50,51^. Sometimes, during geomagnetically disturbed days associated with geomagnetic storms created by solar flares, coronal mass ejections etc., gravity waves generated due to Joule heating of the polar region ionosphere can travel equatorward and reach subtropical regions. As the planetary geomagnetic Ap index was 4 on 15 August 2017, which is considered as a geomagnetically quiet day, the MSTIDs detected on this day over Kashmir region cannot be associated with polar region Joule heating effects of the ionosphere.

In order to study more details of MSTIDs induced by topography generated gravity waves, we analysed one whole year data of 2018. Unfortunately, we found no MSTIDs in this year but on many days mesospheric gravity waves are present in the images associated with 840 nm (~ 85 km height) and 557 nm (~ 97 km height) filters, which indicates that MSTIDs are rare to see in this region of Kashmir even though mesospheric gravity waves are often present. It is to be noted that for the occurrence of MSTIDs, atmospheric gravity waves act only as seed mechanisms out of many other source mechanisms for the generation of MSTIDs. Duly et al.^52^ point out that even a random perturbation in the F region ionosphere can initiate MSTID modes that are consistent with the Perkins instability mechanism and that the growth rate of a specified k perturbation associated with MSTIDs agrees well with linear theory. Dissipation of gravity waves can lead to generation of perturbation in the ionospheric conductivity or electron density. In the present work, we observed south-westward and north-westward propagating gravity waves at the heights of ~ 85 km and ~ 97 km. From raytracing analyses (not shown here), it is found that the background atmospheric wind conditions at these heights are such that they could propagate above their respective heights and hence these would have dissipated in their adjacent higher heights. The dissipation would have led to creation of atmospheric turbulence and that would have been converted into ionospheric turbulence (modulations in the ionospheric conductivity) through ion-neutral collision processes in the lower E region ionosphere. These lower E region ionospheric turbulence and the associated electric field fluctuations would have been communicated to the F region ionosphere through inclined geomagnetic field lines as they are considered as highly conducting equipotential lines^53,54^ or through secondary gravity waves generation from the dissipation of primary waves as discussed above. To determine exactly what would have been possible, it is imperative to have high time (say minutes) and spatial (say hundreds of meters in the vertical and few kilometres in the horizontal directions) resolution simultaneous measurements of atmospheric wind velocities and temperature, ionospheric conductivities, electric field intensity and electron density in the height region of 90–300 km. Without these atmospheric and ionospheric parameters values in this height region, it is never possible to pinpoint the exact causative mechanism of MSTIDs in the midlatitude F region ionosphere.

In brief, it can be said that a random perturbation in the F region ionosphere can result in the development of MSTID modes that are consistent with the Perkins instability and that the growth rate of a specified k perturbation associated with MSTIDs can agree well with linear theory^52^. Two things can happen when primary gravity waves get dissipated in the lower thermosphere region:

(1) Dissipation of primary mesospheric gravity waves can lead to generation of perturbation in the ionospheric conductivity, electric field and electron density. Through E and F region electrodynamical coupling because of the highly conducting equipotential geomagnetic field lines, these perturbations can reach to the F region ionosphere and initiate MSTIDs^53,54^.

(2) Dissipation of primary mesospheric gravity waves can lead to generation of secondary gravity waves that can propagate to the F region ionosphere and particular mode of gravity waves can initiate MSTIDs^8^.

The present study stresses that to understand the exact mechanism of electrodynamical coupling between the E and F region ionosphere, it is must to have experimental facilities to determine high time and space resolution atmospheric winds and temperature and ionospheric conductivity, electron density, electric field.

## Data Availability

While the ERA-5 data can be obtained from the website of https://cds.climate.copernicus.eu/cdsapp#!/dataset/reanalysis-era5-pressure-levels?tab=form, the ISRO-Bhuvan topographic data can be obtained from https://bhuvan.nrsc.gov.in/bhuvan_links.php. The TIDI-TIMED satellite data can be accessed from the website of http://timed.hao.ucar.edu/tidi/data.html. The airglow imager data are available in the institutional repository and they can be accessed via the institute (National Atmospheric Research Laboratory, NARL) website of https://www.narl.gov.in. The complete data archiving in this institute (NARL) website is underway and will be made available to the public in the near future. SAMI 3 model data are available at https://ccmc.gsfc.nasa.gov/models/modelinfo.php?model=SAMI3 (last visited on 16 June 2020).
